# Rehabilitation Nursing for Cerebral Stroke Patients within a Suitable Recovery Empty Period

**Published:** 2017-02

**Authors:** Hu ZHIYAN, Li NIN, Chen BAOYUN, Gong ZUNKE, Wang QINGHONG, Fan LANGE

**Affiliations:** Xuzhou Central Hospital, Xuzhou Recovery Hospital, School of Medical Technology, Xuzhou Medical University, Xuzhou, China

**Keywords:** Recovery empty period, Cerebral stroke, Extended rehabilitation nursing

## Abstract

**Background:**

We aimed to research the value of extended nursing for cerebral stroke patients within a suitable recovery empty period.

**Methods:**

Seventy-two cerebral stroke patients were randomized to a control group or treatment group at the recovery period at Xuzhou Recovery Hospital, China in 2016. A recovery guidance exercise was applied to the control group for a set time, while a recovery guidance exercise combined with functional training were applied to the treatment group within the recovery empty period (at 6:00–7:00 a.m. and 7:00–8:00 p.m.). The recovery effect was compared after three months.

**Results:**

Following the three-month intervention, both the control and treatment groups’ scores for the Fugl-Meyer balance evaluation and the Barthel indicator were increased. There was a statistically significant increase in the treatment group (*P*<0.05). Scores for the Self-Rating Depression Scale in both groups declined and the decline in the treatment group was statistically significant greater when compared to the control group (*P*<0.05). The total depression rate for the treatment group was significantly lower than the control group and the severe extent of depression in the treatment group was significantly less than the control group (*P*<0.05). Both groups’ scores for the PSQI also decreased with a significantly greater increase in the treatment group (*P*<0.05).

**Conclusion:**

Extended nursing within a suitable recovery empty period can improve the patient’s prognosis concerning physical activity and mood.

## Introduction

The recovery empty period refers to the period after sufficiently obtaining food and having a break except conventional fixed training time, which was not scientifically controlled by the diplomate and the nurse (1). Cerebral stroke recovery training is a sustaining process. Suitable training intensity and time can improve the effect of the functional recovery and shorten the training time (2). After cerebral stroke, the recombination and reproduction of the nerve cell impulsion and the synaptic connection are required to repeatedly and mechanically train, and reproduce a positive response through stimulating the injured cells of the body and speech activity (3).

In China, the fine recovery training room is generally conducted from 9:00 – 12:00 a.m. and 2:00 – 4:00 p.m. and the vigor of the patient is relative good at 6:00 – 7:00 a.m. and 7:00–9:00 p.m., but lack in correct and effective training. Currently, there is little research in China on how to conduct recovery training effectively within the recovery empty period.

In response to this dearth of research, our center is researching whether the use of functional strength training and careful observation of a patients’ life routines during the suitable recovery empty period impacts patient outcomes.

## Materials and Methods

### Patient data

Seventy-two cerebral stroke patients were selected from patients entering Xuzhou Central Hospital from June 2014 to June 2015 to receive the function recovery training during the recovery period. Patients were included in the study based on the following criteria: 1) Well compliant, correctly understands the recovery skill, and accurately expresses discomfort; 2) Illness/condition stable within the last month and no new cerebral stroke has occurred; and 3) The recovery training has to be conducted in at least 3 months. The exclusion criteria were: 1) Cerebral stroke is combined with the serious underlying disease such as the dysfunction of the visceral organs such as heart, liver and kidney; 2) Serious mental diseases such as anxiety, depression and suicide; and 3) incompatibility for the questionnaire and function score. The Ethics Committee of Xuzhou Recovery Hospital, China approved the research. Written informed consent was obtained from all participants before the study.

The control group and treatment group each contained 36 participants. There were 20 men and 16 women in the control group. The age range of the control group participants was 42–76 yr old, with an average of 56.8±14.2 yr. The cerebral stroke occurred approximately 1–3 months prior to the intervention, with an average of (1.4±0.5) month. There were 21 men and 15 women in the treatment group. Their age was at a range of 44–75 yr old, with an average of 57.2±13.5. The cerebral stroke occurred approximately 0.5–3 months prior to the intervention, with an average of 1.3±0.6 months. There was no significant difference in the baseline data between the control and treatment group.

### Research methods

The research was completed by the recovery team. The control group participated in the recovery guidance training during two times each day from 9:00–12:00 a.m. and 2:00–4:00 p.m. The body and speech training were conducted on all patients in the recovery training room.

The main methods were as follows: 1)The body activity was conducted at the early period and it included the abduction, internal rotation and upthrow of the upper limb, and the raise, buckling, extorsion and adduction of the lower limbs once per day for 20–30 min; The function training including activities such as dressing clothes, eating foods, holding things and brushing teeth at the later period; 2) The nerve development therapy and the common Bobath technology use the normal post and balance reflex to induce normal action and adjust muscular tension and to establish a normal moving model.

Brunnstrom technology mainly uses the common post occurring to the hemiplegia period to conduct the functional exercises to coordinate the action. Rood technology uses some type of stimulation to cause a response and activate moving functions. All processes should be observed and judged at any time, and regularly conducted according to the reaction of the patient in line with the complexity degree to conduct the higher training. 3) Brain function recombination technology such as the method of relearning from the exercise, in which the recovery of the moving function and ability of the daily life was regarded to be a process of retraining and reforming after the injury of the brain function. Through special guidance and training, some conventional moving function can be practiced such as the body balance and maintaining the walking ability. The compulsory therapy refers to a method for curing the neuronal injury established after the central nervous system of the patient is injured. This therapy can avoid acquisition apraxia and form the recombination of the reliant function. 4) Acupuncture and moxibustion and physiotherapy in Chinese traditional medicine: this method applies merdian-collateral theory, and a suitable technique to conduct the acupuncture and moxibustion through the acupoint selection, and combines with the principle of “Ti, Nian and Zhuan”; the infrared ray physiotherapy is used to heat regionally.

The treatment group participated in function training (6:00–7:00 a.m. and 7:00–8:00 p.m.) during the recovery empty period for 10–15 min. The main content included: 1) check whether the neurological function was injured again, and prevent the complication such as hypostatic pneumonia, bedsore and malnutrition; and 2) understand the recovery progress, achievement and untoward effect; correctly promote the recovery and enhance the confidence and perseverance for training; encourage, recognize and record the better response such as the improvement of the limb muscular strength, the increase of the moving scope and the speech function, and provide the feedback to the physical therapist, who is in favor of adjusting the training solution timely and encouraging the patient to actively coordinate with the training; timely understand the untoward effect in the training such as excessive training strength and time which can be solved by reducing the difficulty level of the training, and reasonably decreasing the training time, and increasing the training frequency; without reducing the training effect, improve the training coordination and comfort degree for the patients to a certain degree; and 3) conduct the rehabilitation nursing for, such as removing the body position of the hemiplegia or paraplegia patient on the bed, and conducting the self-cleaning training and eating and drinking, which supplements the work of the rehabilitation therapist greatly.

### Observation indicator

The research team assessed the motor function of patients using the Fugl-Meyer balance evaluation sheet. The Barthel indicator was used for evaluating the ability of daily living. Self-Rating Depression Scale (SDS) was used for evaluating the mental state and Pittsburgh Sleep Quality Indicator (PSQI) for sleep quality.

The Fugl-Meyer balance evaluation sheet includes 7 items, 3 sitting positions and 4 standing positions, each item includes 3 levels, 0, 1, 2 score(s) respectively. Zero represents it fails to complete the motion, 1 represents it completes the motion and 2 represents it fully complete the regulated motion. As to the Barthel indicator, 100 scores means Grade A - normal, 60–100 scores means Grade B – well (self-care basically), 30–60 scores means Grade C - mild dysfunction (need help), 10–30 scores means Grade D - severe dysfunction (obviously rely on) and <10 score (s) means Grade E – disability (completely rely on). SDS includes 2 items of psychotic emotional symptoms, 8 items of corporality disorders, 2 items of psychomotor disorders and 8 items of depressed psychological disorders, the upper limit of the total raw scores is 41 scores, the lower the score is, the better the state is. The standard score is the integral part of the result after the raw score multiplies by 1.25, in case the SDS standard score is more than 50, then it is depressive symptom. The severity of depression equals accumulated points of each item/80, below 0.5 indicates there is no depression, 0.5–0.59 indicates there is slight to mild depression, 0.6–0.69 indicates there is medium to severe depression, and more than 0.7 indicates there is severe depression. PSQI is composed by 9 questions, the first 4 questions are sentence completion, and the remaining questions are choice questions, among which the fifth question includes 10 small items. Each question is classified by Level 0–3, the score of accumulated items is the total score of PSQI, the range of total score is 0–21, the higher the score is, and the worse the sleep quality is. It will take 5–10 minutes for the participant to complete the answer.

### Statistical methodology

SPSS19.0 (Chicago, IL, USA) was used for data analysis. The quantitative data was represented by the mean value ± standard deviation. The comparison among the groups was conducted by independent-samples T test and the comparison within the groups was conducted by paired T test. The qualitative data was represented by case number or (%). The comparison among the groups was inspected by χ^2^ and the ranked data was inspected by sum of ranks. A *P* value of <0.05 indicated that the difference was statistically significant.

## Results

### The comparisons between the score of Fugl-Meyer and Barthel

With regard to the comparison of scores between Fugl-Meyer and Barthel before the intervention, there was no statistically significant difference (*P*>0.05) before the intervention. Both scores for Fugl-Meyer and Barthel increased 3 months after intervention. There was a greater increase in the treatment group. The difference was statistically significant (*P<*0.05) (Table 1).

**Table 1:** Comparison the Fugl-Meyer and Barthel scores in the treatment and control groups

**Groups**	**Fugl-Meyer**		**Barthel**	
	**Before the intervention**	**After 3 months**	**Before the intervention**	**After 3 months**
Control group	4.2±1.2	7.3±1.6	24.2±3.3	75.5±9.0
Treatment group	4.1±1.3	10.5±2.0	23.6±3.5	84.6±9.3
T test	0.632	7.526	0.326	8.625
*P* value	0.748	0.012	0.452	0.007

### The comparison of SDS scores

When comparing the two group’s SDS scores there was no statistically significant difference (*P*>0.05) before the intervention.

After the intervention, both group’s SDS scores declined, and the decline of treatment group was greater with a statistically significant difference (P<0.05). The total depression rate of the treatment group was lower than the control group. This difference was statistically significant (P<0.05) (Table 2).

**Table 2:** Comparison of SDS scores in the treatment and control groups

**Groups**	**Case number**	**SDS before the intervention**	**SDS after 3 months**	**No depression (n(%))**	**Mild depression (n(%))**	**Medium/severe depression (n(%))**	**The total depression rate (n(%))**
Control group	36	66.3±6.3	55.7±8.2	15 (41.7)	10 (27.8)	11 (30.6)	21 (58.3)
Treatment group	36	66.2±6.4	52.4±8.3	24 (66.7)	9 (25.0)	3 (8.3)	12 (33.3)
t		0.629	6.527				
χ^2^					6.701		4.531
*P* value		0.865	0.029		0.035		0.033

### The comparison of PSQI scores

The comparison of two group’s PSQI scores demonstrated no statistically significant difference (*P*>0.05) before the intervention. After the intervention, both group’s PSQI scores declined with a greater decline in the treatment group (*P<*0.05) (Table 3).

**Table 3:** Comparison of PSQI scores in the treatment and control groups

**Groups**	**Before the intervention**	**After 3 months**
Control group	17.2±4.3	13.6±3.2
Treatment group	17.5±4.5	8.5±3.0
T test	0.524	7.532
*P* value	0.869	0.006

## Discussion

This study demonstrates that choosing the suitable rehabilitation intensity that patients can tolerate is an important aspect of improving effects of rehabilitation. After adding 67% rehabilitation time, the improvement of illness state was better, and the average length of stay was reduced by 14 days (4). With regard to the stroke patients in the acute stage and sub-acute stage, there was positive correlation between the improvements of motor function and duration of rehabilitation cure (5). Hendricks et al. (6) conducted a meta-analysis of 174 articles, and they found that complete rehabilitation rate of motor function was about 15%, the rehabilitation time of the patients with severe illness was double, 95% patients would recover to different degree within 6 months of illness, and it could reach 100% basically at the end of one year.

At present, the new technologies of neurological rehabilitation include Bobath, Brunnstrom, Rood, Motor Relearning Program (MRP), Constraint-induced Movement Therapy (CMT) and training method by instruments etc. (7). In addition, there is comfortable therapy (8). This is a kind of psychological persuasion method aimed at psychological illnesses, such as negative emotions, being agitated and world-weary and other condition by use of psychological fluctuation of patients during the period of illness. It can make the patients feel relaxed psychologically, rekindle the hope for the life, develop the confidence to actively cooperate with rehabilitation, accept the facts with a peaceful mind, be brave to pour out feelings to the caregivers and listen to the useful advice. The operation effect within the vacuum recovery period is better. The functional exercises train patients by2 guiding their daily actions, such as feed, wearing and arrangement to motivate the motor function (9). The bio-feedback technology, operation therapy method, general intelligent rehabilitation system and others also show good effects (10).

In this study, the methodology included full use of ward vacuum period, identifying two suitable periods by comparison and evaluation, namely 6:00–7:00 a.m. and 7:00–8:00 p.m. Because the rehabilitation patients would conduct rehabilitation training in the therapeutic gymnastic room during daytime, it would consume a large amount of energy most of the time, and the patients needed rest afterwards and go back to their ward at noon. Training was conducted after participants regained their energy. In cases where the research team gave ward extended guidance for the patients immediately after he/she went back to the ward, then the patients and their families would be unwilling to accept it due to fatigue. The research team would get half the result with twice the effort in case of guiding and educating at this time, and it would yield twice the result with half the effort (11–12) when there was guiding after the patients getting up, having a good mental state and completing the physical exercise therapy, having dinner and resting, and they were easy to accept it. Administrative staff arranged a nurse to provide nursing care, give basic rehabilitation guidance at the working hours, namely 6:00–8:00 a.m., 6:00–9:00 p.m., and understands the conditions of patients in the therapeutic gymnastic room one day and gives targeted professional guidance.

## Conclusion

Both the scores of Fugl-Meyer and Barthel increased after the 3-month intervention, and improvement in the treatment group was greater. Both group’s SDS scores declined after the intervention, and the decline of the treatment group was greater; the total depression rate of the treatment group was lower than the control group, and the depression rate was slightly better, and both group’s PSQI scores declined after the intervention with the decline of treatment group being greater, and both differences were statistically significant. Conducting the rehabilitation training fully, efficiently and scientifically using the rehabilitation vacuum period may not only provide a viable and shortened rehabilitation treatment course, but also reduce the economic cost of rehabilitation. In general, extended nursing during a suitable time within the rehabilitation vacuum period could improve the patient’s prognosis.

## Ethical considerations

Ethical issues (Including plagiarism, informed consent, misconduct, data fabrication and/or falsification, double publication and/or submission, redundancy, etc.) have been completely observed by the authors.
